# Heart Failure and Edema Costs in Patiromer and Sodium Zirconium Cyclosilicate Users

**DOI:** 10.34067/KID.0000000000000483

**Published:** 2024-06-05

**Authors:** Nathan Kleinman, Jennifer Kammerer, Charuhas Thakar

**Affiliations:** 1 Kleinman Analytic Solutions, LLC, Paso Robles, California; 2 CSL Vifor, Redwood City, California; 3 Division of Nephrology and Hypertension, University of Cincinnati, Cincinnati, Ohio

**Keywords:** economic analysis, economic effect, electrolytes, heart failure, hospitalization, outcomes

## Abstract

**Key Points:**

- Prior research suggests differences in rates of heart failure hospitalization or serious emergency department visits between patients on patiromer versus sodium zirconium cyclosilicate.
- Total costs of heart failure–related hospitalizations and emergency department visits may be lower in patients on patiromer compared with sodium zirconium cyclosilicate.

**Background:**

Previous work suggested differences between patients taking patiromer or sodium zirconium cyclosilicate (SZC) in real-world risk of heart failure (HF) hospitalizations and edema hospitalizations or emergency department (ED) visits (edema events). We further investigated these differences to assess economic importance. Retrospective study using published event rates and mean costs derived from Optum's deidentified Clinformatics Data Mart Database.

**Methods:**

We designed a model to estimate adjusted economic offsets that combined respective patiromer and SZC HF hospitalization (25.1 and 35.8; difference 10.7 [95% confidence interval (CI)^2^, 2.6 to 18.8]) and edema event (3.4 and 7.1; difference 3.6 [95% CI, 1.7 to 7.1]) rates/100 person-years from the original published work with costs from our parallel data extract spanning 2019–2021, adjusted to 2021 US dollars.

**Results:**

In a base case of mean HF hospitalization, edema event, and 30-count potassium-binder prescription costs from our data extract, the estimated mean savings with patiromer was $1428 per person per year (95% CI, −$1508 to $4652). Respective costs per person per year for patiromer versus SZC were $8526 versus $12,622 (difference $4096 [95% CI, $116 to $7320]) for HF hospitalization and edema events, and $10,649 versus $7981 (difference −$2668) for potassium binders, totaling $19,175 for patiromer versus $20,603 for SZC.

**Conclusions:**

With differing drug costs, hospitalization and ED costs offset this difference when event rates were numerically small. Model outcomes were driven by HF hospitalization cost and least influenced by edema ED visit cost. A limitation was that the Clinformatics Data Mart data extract may differ from the original work.

## Visual Abstract

**Figure:**
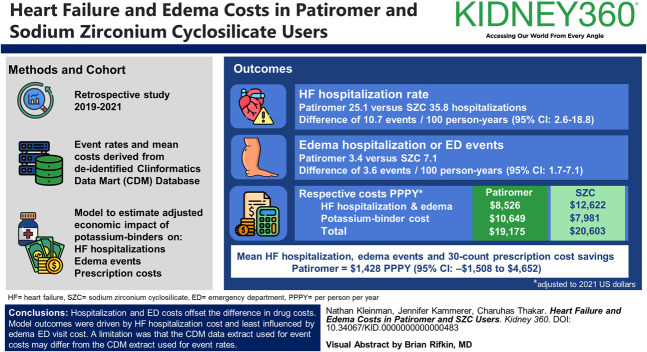

## Introduction

Hyperkalemia (serum potassium >5.0 mmol/L) occurs when the excretion of renal potassium is limited by reductions in GFR, distal sodium delivery, tubular flow, or the expression of aldosterone-sensitive ion transporters in the distal nephron.^1–3^ Major risk factors that may lead to hyperkalemia include kidney disease, adrenal disease, diabetes mellitus, and the use of renin-angiotensin-aldosterone system inhibitors (*e.g*., angiotensin-converting enzyme inhibitors, angiotensin receptor blockers, aldosterone receptor antagonists, and direct renin inhibitors).^1,2^ Patients with CKD and patients who are on dialysis are particularly predisposed to hyperkalemia.^1,4^ The consequences of hyperkalemia may include cardiac dysrhythmias and death.^1,4,5^

Patiromer and sodium zirconium cyclosilicate (SZC) are potassium binder medications approved to treat non–life-threatening hyperkalemia; both have been shown to be efficacious in lowering and achieving potassium levels within normal limits.^5–12^ Patiromer was approved by the US Food and Drug Administration in 2015 and uses calcium as the counter-ion exchanger when binding free potassium ions in the gastrointestinal tract.^13,14^ SZC was approved by the US Food and Drug Administration in 2018 and exchanges hydrogen and sodium for potassium in the gastrointestinal tract.^13,14^

The availability of such agents has allowed clinicians to continue treatment with angiotensin-converting enzyme inhibitors and angiotensin receptor blocker agents, while treating the complication of hyperkalemia through potassium exchange with either calcium or sodium.^15^ Heart failure (HF) and worsening edema are common comorbidities in patients with CKD and hyperkalemia. Thus, on one hand, these agents may facilitate cardiovascular risk reduction by allowing use of renin-angiotensin-aldosterone system–blocking therapies, but with potential compound unwanted events that may be drug-related or class-related (*i.e*., worsening of gastrointestinal motility and related adverse events, hypokalemia, hypomagnesemia, fluid overload/edema, intestinal necrosis). For instance, SZC is associated with an increased likelihood of edema, likely due to increased systemic sodium absorption.^11,14^ Approved labeling states that each 5 g dose of LOKELMA (SZC) contains approximately 400 mg of sodium, but the extent of absorption by the patient is unknown. While taking SZC, patients should be advised to monitor for edema and/or adjust dietary sodium.^16^ Patients who take SZC and have low tolerance for small increases in sodium—such as patients with HF, hypertension, and CKD—may be susceptible to adverse outcomes.^13^ The most common adverse reactions (≥2%) reported in the VELTASSA (patiromer label) are constipation, hypomagnesemia, diarrhea, abdominal discomfort, and flatulence.^17^

A recent study by Zhuo *et al.* compared rates of severe edema events and rates of HF hospitalization in propensity score–matched cohorts of patients with newly prescribed patiromer (*N*=2839) or SZC (*N*=1126).^13^ The rate of severe edema (defined as a hospitalization or emergency department [ED] visit with a diagnosis of edema in any position) was significantly higher in the SZC cohort (7.1 events per 100 person-years) than in the patiromer cohort (3.4 events per 100 person-years; difference [95% confidence interval (CI)^2^]=3.6 [1.7 to 7.1]). For HF hospitalizations, the incidence rate was significantly higher in the SZC cohort (35.8 hospitalizations per 100 person-years) than in the patiromer cohort (25.1 hospitalizations per 100 person-years; difference [95% CI]=10.7 [2.6 to 18.8]).

With numeric differences in HF hospitalization and severe edema event rates, determining the total cost effect must account for both event-related costs and differences in potassium-binder drug costs. Information on such cost offsets has not been published previously and may contextualize clinical and economic relevance for event differences because it relates to treatment of hyperkalemia. The objective of the current study is to obtain average costs of HF hospitalizations and edema events from Optum's deidentified Clinformatics Data Mart (CDM) Database and to apply those cost averages to the differences in event rates found by Zhuo *et al.*^13^ to produce an estimate of the difference in total costs associated with HF hospitalizations and edema events in patients taking patiromer and patients taking SZC.

## Methods

Zhuo *et al.* used CDM as the source for HF hospitalization and edema-related event rates. We used a parallel but distinctly licensed extract from CDM, spanning 2019–2021 dates, to identify mean costs for HF hospitalizations, edema hospitalizations, edema ED visits, and 30-count prescriptions for patiromer and SZC. In general, CDM contains inpatient, outpatient, and professional service medical claims and prescription claims for more than 75 million individuals enrolled in commercial or Medicare Advantage health insurance plans in the United States.

This retrospective, administrative claims database analysis was based on historic deidentified patient data and did not involve patients directly; therefore, institutional review board/ethics committee approval was not necessary or applicable.

Zhuo *et al.* selected International Classification of Diseases, Tenth Revision, Clinical Modification (ICD-10) diagnosis codes with the first three digits I50 in any position within the claim record to identify HF hospitalizations. ICD-10 codes beginning with R60 in any position were used to identify edema hospitalizations and ED visits. We used the same ICD-10 codes as in Zhuo *et al.* to identify average costs of HF and edema hospitalizations and ED visits from our parallel extract of the CDM data.

The average event-related costs were adjusted for inflation to 2021 US dollars using the hospital services consumer price index. The cost values from CDM included both professional and facility fees and covered both payments made by the insurer as well as out-of-pocket (OOP) deductible, coinsurance, and copay payments made by the patient.

Then, the average CDM HF hospitalization cost was multiplied by the rate of HF hospitalizations in patients taking patiromer or SZC as published in Zhou *et al.*^13^ to determine the estimated total cost of HF hospitalizations per 100 person-years in each cohort. Similarly, the average CDM cost of edema hospitalizations or ED visits (weighted by proportion of edema hospitalizations versus ED visits) was multiplied by the rate of edema events in patients taking patiromer or SZC as published in Zhuo *et al.*^13^ to identify the estimated total cost of edema events per 100 person-years in each cohort. The 95% CIs for estimated costs were obtained by multiplying the Zhuo *et al.* event rate 95% lower and upper confidence limits by the average CDM event costs. These rates were then divided by 100 to express the rates on a per person per year (PPPY) basis.

PPPY medication costs were calculated from 2021 prescription claims data by multiplying the average cost of a 30-count prescription of any dose of either patiromer or SZC by 12 (assuming a full year of medication). The medication costs included patient OOP and plan-paid amounts but did not reflect plan rebates.

Total estimated PPPY costs for each cohort were calculated by totaling the estimated HF hospitalization costs, edema event costs, and medication costs. All queries and calculations were performed in Microsoft Structured Query Language Server and Microsoft Excel.

## Results

Table 1 shows the number of events and average cost per event from our CDM extract, along with event rates and 95% CIs for the patiromer and SZC cohorts as published in Zhuo *et al.*; the number and average cost of 30-count patiromer and SZC prescriptions from our CDM extract are also provided. Components in Table 1 are used as inputs to the cohort cost model.

**Table 1:** Heart failure hospitalization and edema event cost model inputs

Model Inputs	*N*	Average Cost Per Event
HF hospitalizations^a^	2,095,607	$31,186
Edema hospitalizations^a^	514,859	$37,651
Edema ED visits^a^	441,342	$552

	Patiromer	SZC
HF hospitalization rate/100 person-years^b^	25.1	35.8
Rate difference (95% CI)^b^	10.7 (2.6 to 18.8)	
Edema event rate/100 person-years^b^	3.4	7.1
Rate difference (95% CI)^b^	3.6 (1.7 to 7.1)	
No. of 30-count prescription fills^a^	34,578	13,181
Average cost per prescription^a^	$887.44	$665.11

CDM, Clinformatics Data Mart; CI, confidence interval; ED, emergency department; HF, heart failure; SZC, sodium zirconium cyclosilicate.

a From current Clinformatics Data Mart extract based on 30-count claims and all doses.

b From the study by Zhuo *et al.*.^13^

After multiplying cost averages by event rates and dividing by 100, Table 2 shows event, medication, and total estimated costs PPPY for the patiromer and SZC patient cohorts.

**Table 2:** Heart failure hospitalization and edema event cost model outputs

PPPY	Patiromer	SZC	Difference	95% Confidence Range
HF hospitalizations	$7827.60	$11,164.47	$3336.87	$810.83 to $5862.90
Edema hospitalizations	$689.29	$1439.39	$750.11	$344.64 to $1439.39
Edema ED visits	$8.66	$18.09	$9.43	$4.33 to $18.09
Total event costs	$8525.55	$12,621.95	$4096.40	$1159.80 to $7320.39
Medication costs	$10,649.28	$7981.32	−$2667.96	—
Total costs	$19,174.83	$20,603.27	$1428.44	−$1508.16 to $4652.43

ED, emergency department; HF, heart failure; PPPY, per person per year; SZC, sodium zirconium cyclosilicate.

**Figure 1:**
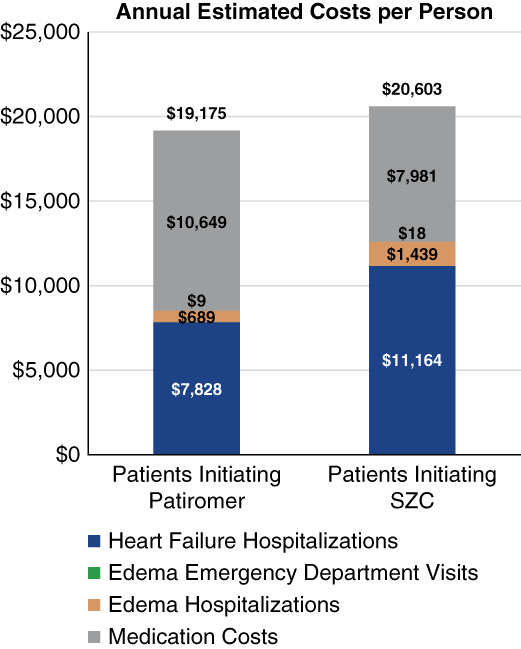
**Annual estimated costs per person patiromer cohort estimated costs are $1428 lower per person than SZC cohort costs.** SZC, sodium zirconium cyclosilicate.

The estimated cost of HF hospitalizations and edema events PPPY is $4096.40 (48.0%) higher in the SZC cohort than in the patiromer cohort. Applying the event cost averages to the 95% CIs for the Zhuo *et al.* study event rates, we found that estimated event costs are significantly higher in the SZC cohort than in the patiromer cohort. After adding medication cost, the total estimated costs (events+medications) PPPY are $1428.44 (7.4%) higher in the SZC cohort than in the patiromer cohort (Figure 1). Because the CI for the estimated total costs includes zero, the $1428.44 estimated difference is not statistically significant.

## Discussion

Our study found that numeric differences in HF hospitalization and edema events reported by Zhou *et al.* merit economic consideration based on an approach that multiplied respective event rates by average costs per HF hospitalization ($31,186), edema hospitalization ($37,651), and edema ED visit ($552). The overall event cost PPPY was $4096 higher in patients taking SZC than in patients taking patiromer. After accounting for medication cost, there was a PPPY trend showing $1428 higher costs in patients taking SZC than in patients taking patiromer; however, this did not reach statistical significance. This study seems to be the first such analysis estimating total costs of care after offsetting potassium-binder drug costs.

Several studies were found in the literature that reported average cost per HF hospitalization.^18–21^ After adjusting their findings for inflation to 2021 dollars, these average costs per hospitalization ranged from $14,999 to $21,387. The source of these hospitalization costs was the Healthcare Cost and Utilization Project,^22^ which does not include professional fees or most patient-paid OOP costs. In addition, most of these studies used hospitalizations with HF as the primary diagnosis instead of any diagnosis.

Several published studies used a methodology similar to that used in this study; namely, multiplying the average cost per event by the event rate in two distinct populations and then comparing the resulting average cost per person in the two populations.^23–25^

### Limitations

This study has several limitations. We acknowledge that this analysis is focused on determining drug costs and costs of care in totality on the basis of real-world data, and we did so by choosing a prior peer-reviewed manuscript in the literature by Zhou and colleagues. Although the study of Zhuo *et al.* also did use CDM data, their specific data extract was not available to us, and there may be differences between their extract and ours, including the time span for claims-based event capture. Hence, we recapitulated similar ICD-10–based diagnosis codes from the same data source and added costs of care and drug costs from contemporaneous dataset. We understand that the CDM data are from a single payer and formulary, which may influence prescriber preference, drug prices, and patient access. In addition, medication costs were not dose-specific nor were event rates dose-adjusted. SZC patients taking 15 g must take two packages, which likely increases SZC medication cost and the model's reported cost difference. However, we attempted to use costs for all doses for a 30-day period and then annualize the costs to per patient per year. Claims data may not fully characterize and capture HF or edema events and do not measure the timing or levels of actual sodium changes.

As previously reported (cite the Zhou *et al.* rather than write the name here in conclusions) rates of HF hospitalizations, edema hospitalizations, and ED visits in patients taking patiromer were numerically lower versus patients taking SZC. This study applies the average costs of these events to the rate differences and shows that patiromer patients had $4096 lower event costs PPPY than SZC patients. Even after factoring in medication costs, total costs trended lower in patients taking patiromer. In addition to the clinical benefits of fewer hospitalizations and ED visits, patiromer may affect economic outcomes to be considered in decision making about potassium binder selection. Future research should examine the costs of these medications as well as the rates and costs of hospitalizations and ED visits in a multipayer dataset and should include assessment of temporal association with sodium changes. Effect on key quality measures may also be of future research interest.

## Data Availability

Previously published data were used for this study. Partial restrictions to the data and/or materials apply. Some results from a study by Zhuo et al were used in this study. Citation: Zhuo M, Kim SC, Patorno E, Paik JM. Risk of Hospitalization for Heart Failure in Patients With Hyperkalemia Treated With Sodium Zirconium Cyclosilicate Versus Patiromer. J Card Fail. 2022;28(9):1414-1423. Mean costs were derived from Optum’s de-identified Clinformatics Data Mart (CDM) Database.
